# Gangrenous cholecystitis in an asymptomatic patient found during an elective laparoscopic cholecystectomy: a case report

**DOI:** 10.1186/1752-1947-5-199

**Published:** 2011-05-21

**Authors:** Sunil Chaudhry, Rima Hussain, Rajaganeshan Rajasundaram, David Corless

**Affiliations:** 1 Department of Surgery, Leighton Hospital/NHS, Crewe, CW1 4QJ, UK; 2 Department of Histopathology, Royal Victoria Infirmary/NHS, Newcastle Upon Tyne, NE1 4LP, UK; 3 Department of Surgery, Leighton Hospital/NHS, Crewe, CW1 4QJ, UK

## Abstract

**Introduction:**

Gangrenous cholecystitis is a severe complication of acute cholecystitis. We present an unusual case of gangrenous cholecystitis which was totally asymptomatic, with normal pre-operative parameters, and was discovered incidentally during a laparoscopic cholecystectomy. We have not found any similar cases in the published literature.

**Case presentation:**

A 79-year-old British Caucasian man presented initially with acute cholecystitis which responded to conservative management. After six weeks he was asymptomatic and had normal blood parameters. An elective laparoscopic cholecystectomy was performed and our patient was found to have a totally gangrenous gall bladder.

**Conclusion:**

It is important to keep a high index of suspicion for the diagnosis of gangrenous cholecystitis in order to avoid potentially serious complications.

## Introduction

Gangrenous cholecystitis (GC) is a serious complication of acute cholecystitis [1,2]. It is the result of marked distension of the gallbladder causing increased tension in the gallbladder wall. Associated inflammation leads to ischemic necrosis of the wall, with or without associated cystic artery thrombosis [3]. It is more common in men and in patients with co-existing cardiovascular disease and leukocytosis (white cell count (WCC) greater than 17 × 10^9^/L) [3]. Other associated factors include diabetes, critical illness and a high C-reactive protein (CRP) level [4]. Pre-operative diagnosis of this condition may prove difficult. Once suspected, patients with GC generally undergo emergency cholecystectomy in order to avoid life-threatening complications [3].

## Case presentation

A 79-year-old British Caucasian man with a known history of diabetes mellitus was admitted with severe right upper abdominal pain of one week's duration, which worsened on the day of admission and was associated with nausea and vomiting.

On physical examination our patient was apyrexial and hemodynamically stable. An abdominal examination revealed marked tenderness in his right upper quadrant. Laboratory investigations on admission showed a WCC 23.7 × 10^9^/L, CRP 148 mg/L, alkaline phosphatase 54 IU/L, alanine aminotransferase 31 IU/L, bilirubin 12 mmol/L, amylase <30 IU/L and oxygen saturation on air of 95%.

He was treated for presumed acute cholecystitis and was started on co-amoxiclav, 1.2 g taken intravenously three times a day.

The day after his admission, an abdominal ultrasound scan revealed a gallbladder-shaped echogenic viscous fluid present in his gallbladder fossa with posterior acoustic shadowing. An unenhanced computed tomography (CT) scan of his abdomen taken on the same day showed his gallbladder to be slightly distended, with a few small calcific stones. However, the wall of the gallbladder did not appear thickened. No intra-hepatic or extra-hepatic duct dilatation was seen. There was a small rim of fluid anterior to the surface of the right lobe of his liver, between his gallbladder and duodenum, which was suggestive of acute cholecystitis.

Our patient responded well to the intravenous antibiotics, and after four days his blood counts had returned to normal (WCC 7.1 × 10^9^/L; neutrophils 5.05 × 10^9^/L). He was discharged home after five days, with a prescription for oral co-amoxyclav for five more days. An elective laparoscopic cholecystectomy was performed six weeks after discharge, by which time our patient had no abdominal pain and was otherwise well. Pre-operative blood test results were normal.

**Figure 1:**
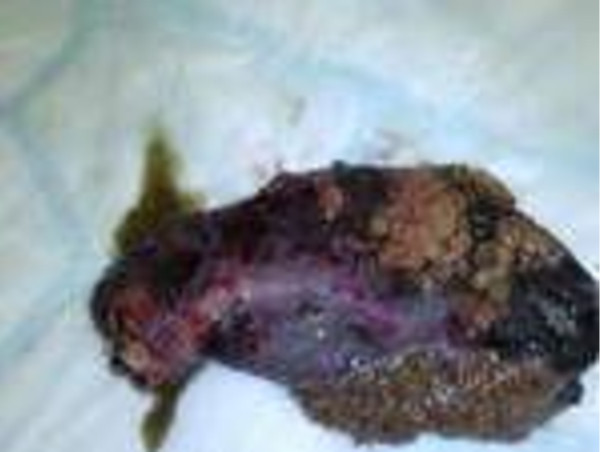
**Gangrenous cholecystitis**.

**Figure 2:**
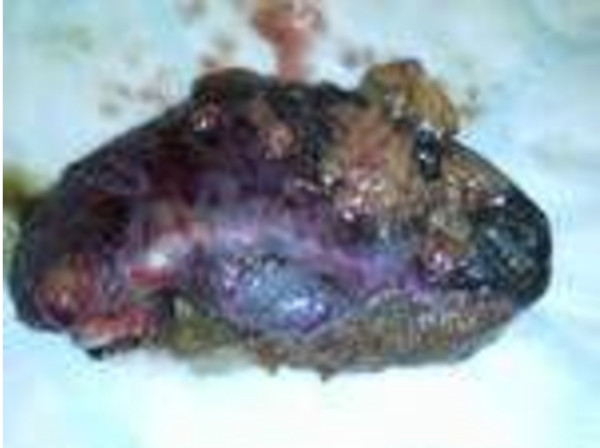
**Gangrenous cholecystitis**.

At the laparoscopy, the fundus of his gallbladder was found to be gangrenous and covered by adherent omentum. The omental adhesions were gently freed revealing an entirely gangrenous gallbladder, which was thick, black and distended. Forty milliliters of dark hemorrhagic fluid were aspirated to aid manipulation. The whole gallbladder was gangrenous up to the cystic duct (Figures 1 and 2). The Calot's triangle was dissected, and after division of the cystic duct and artery, clips were applied to the viable duct and artery. The procedure was completed uneventfully and a 14F drain left in the gallbladder fossa.

Our patient was well post-operatively and kept on antibiotics for five days. He was discharged uneventfully on the sixth day after the operation.

## Discussion

GC is the last stage of gall bladder inflammation [4] and, in spite of its grave prognosis, its diagnosis can be elusive, both clinically and on laboratory investigation. The incidence of GC ranges from 2% to 29.6% in all patients with acute cholecystitis, in various surgical series, and generally occurs in older patients [3]. Many factors have been implicated in its formation. Fagan *et al*. [5] demonstrated that nine variables were associated with GC, but Contini *et al*. [4], showed that there is no single clinical or laboratory finding, apart from a high WCC, predictive of severe inflammation of the gallbladder.

Contini *et al*. [4] showed that the time of hospitalization delay plays a crucial role in the formation of GC. The time between the onset of symptoms and hospital admission was significantly longer in patients with GC. The patient's history (timely or delayed admission) and physician's attitude (general practitioner and/or surgeon) are likely to play a role in the progression towards a severe necrosis of the gallbladder wall [4].

Ultrasonography usually serves as the first-line imaging modality for the evaluation of patients with clinically suspected acute cholecystitis. However, CT can play an important role in evaluation of these patients if sonography is inconclusive [3]. The hallmark on sonography of GC is the presence of heterogeneous or striated thickening of the gallbladder wall, which is often irregular with projections into the lumen and pericholecystic fluid collections. The presence of intra-luminal membranes representing desquamative gallbladder mucosa is a specific finding but it is less common [3]. The accuracy of pre-operative ultrasound in diagnosing GC remains uncertain. Twenty-eight percent of patients with GC had ultrasound reports that failed to show any evidence of acute inflammation. This was mainly due to the absence of sonographic Murphy's sign and gallbladder walls of less than 3 mm, both important radiological signs of acute inflammation of the gall bladder [6].

The CT findings most specific for acute GC are gas in the wall or lumen, intra-luminal membranes, an irregular wall and pericholecystic abscess. GC is associated with a lack of mural enhancement, pericholecystic fluid and a greater degree of gallbladder distension and wall thickening [3].

Our patient is unique because he was asymptomatic and his biochemical investigations prior to elective surgery were normal, which is in contrast to those reported in the literature to date. There is a controversy regarding the best surgical approach to GC with some authors, such as Eldar *et al*. [1], recommending open cholecystectomy for most men over 60 years of age who have significant co-morbidity, large bile stones and elevated bilirubin level. In contrast, Hunt and Chu [1] indicated that laparoscopic cholecystectomy can be used relatively safely and successfully in patients with gangrenous cholecystitis, reporting a success rate of 91% without increased morbidity and no mortality. Others suggested that a more reasonable approach would be an initial examination with the laparoscope, not wasting more than a few minutes to determine whether a dissection would be possible [1]. In the hands of an experienced laparoscopic surgeon, an initial attempt at laparoscopic cholecystectomy is possible, converting to open procedure if necessary. A conversion rate higher than that for simple acute cholecystitis or symptomatic cholelithiasis is to be expected. However, when successful, laparoscopic cholecystectomy is associated with a significantly better outcome and a shorter hospital stay [7]. The conversion rates range from 8% to 75% [3].

GC has a mortality rate of up to 22% and a complication rate of 16-25%. Complications associated with GC include perforation, which has been reported to occur in as many as 10% of cases of acute cholecystitis. Perforation of the gall bladder can then lead to abscess formation or peritonitis. Hence, in contrast to acute cholecystitis, it is important both to diagnose and surgically treat GC prior to complication and/or perforation to avoid its high morbidity and mortality rate [2].

## Conclusion

When dealing with patients with acute cholecystitis, a high index of suspicion is essential for the early diagnosis and treatment of GC. The possibility of a patient, especially an elderly patient with acute cholecystitis, progressing to GC should always considered, even in an apparently improving patient and in spite of the absence of any firm clinical or laboratory findings. The radiological investigations may not be conclusive. There is a need for an early (if not urgent) surgical intervention in acute cholecystitis (whether laparoscopic or open surgery) in order to decrease the time elapsed from the start of symptoms to admission and treatment.

## Abbreviations

CRP: C-reactive protein; CT: computed tomography; GC: gangrenous cholecystitis; WCC: white cell count.

## Consent

Written informed consent was obtained from the legal guardian of the patient for publication of this case report and any accompanying images. A copy of the written consent is available for review by the Editor-in-Chief of this journal.

## Competing interests

The authors declare that they have no competing interests.

## Authors' contributions

RH participated in writing up the case report. SC performed the operation, provided the pictures and revised the case report. RR assisted in the operation and revised the case report. DC (consultant in charge of this patient) participated in writing and revising the case report. All the authors read and approved the final manuscript.
